# Molecular epidemiology of potential candidate markers for chloroquine resistance in imported *Plasmodium vivax* malaria cases in Iran

**DOI:** 10.1186/s12936-023-04553-y

**Published:** 2023-04-10

**Authors:** Sakineh Pirahmadi, Shima Afzali, Akram Abouie Mehrizi, Abbasali Raz, Ahmad Raeisi

**Affiliations:** 1 Malaria and Vector Research Group (MVRG), Biotechnology Research Center (BRC), Pasteur Institute of Iran, Tehran, Iran; 2 National Programme Manager for Malaria Control, Ministry of Health and Medical Education, Tehran, Iran

**Keywords:** Malaria, Mdr1 protein, *Plasmodium vivax*, Drug resistance, Chloroquine, Iran

## Abstract

**Background:**

The spread of *Plasmodium vivax* strains resistant to chloroquine (CQ) has posed a challenge to control strategies aimed at eliminating malaria. Molecular analysis of candidate resistance markers is very important for monitoring the *P. vivax* resistance to CQ in different endemic regions. In the present study, the *multidrug resistance 1* (*pvmdr1*) gene, a possible marker for CQ resistance in *P. vivax*, was evaluated by molecular methods.

**Methods:**

A simple PCR–RFLP method was developed for mutation analysis in *pvmdr1* gene. A number of 120 blood spots were obtained from patients with *P. vivax* mono-infection in 2021. All of the samples were collected from Pakistani patients who travelled to Iran.

**Results:**

None of the samples had any mutation at codon 976 of *pvmdr1*, while the 1076 mutation was detected in 96.2% of the examined isolates. Only two *pvmdr1* haplotypes were identified, including the single mutant (Y976/1076**L**) as the most prevalent haplotype (with 96.2% frequency) and the wild type (Y976/F1076; with 3.8% frequency).

**Conclusions:**

In this study, the major CQ resistance-mediating mutation and multiple mutant haplotypes of the *pvmdr1* gene was not detected. However, continuous monitoring of drug resistance markers and close supervision of the efficacy of CQ is essential to detect the potential emergence of CQ-resistant *P. vivax* isolates in Iran. This data is important for performing future epidemiological surveillance to monitor CQ resistance in this endemic area and the bordering regions.

## Background

*Plasmodium vivax* is the most widely distributed human malaria parasite and the dominant species outside of Africa. The estimated annual global burden of *P. vivax* is about 14.3 million clinical infections [1]. Most tropical regions in the Southeast Asia, Middle East, and the Western Pacific account for about 80–90% of vivax malaria cases outside of Africa [2]. Although it was historically considered to cause benign infection, severe cases and morbidity due to *P. vivax* have notably been reported [3]. Several factors associated with *P. vivax* infection; includin*g* the presence of dormant forms in the liver, the early production of gametocytes, and also the emergence of chloroquine (CQ) resistance, impose major challenges for malaria control programmes.

For decades, CQ has been the mainstay of treatment of vivax malaria. In combination with primaquine, it is very effective against the acute disease and the hypnozoites. However, this status is progressively threatened by the spread of CQ-resistant *P. vivax*. The first report of *P. vivax* resistance to CQ was published in 1989 in Papua New Guinea [4]. Since then, CQ-resistant *P. vivax* has been reported from different endemic countries in Southeast Asia, South Asia, the Middle East, the Americas, and the East Africa [5, 6]. Since CQ-resistant *Plasmodium falciparum* has spread throughout malaria-endemic regions and taken many lives, it seems likely that the same situation will occur in near future with *P. vivax*. Therefore, to avoid the occurrence of the same scenario, extensive research must be done to evaluate the status of CQ resistance. Unlike *P. falciparum*, detection of CQ-resistant *P. vivax* is extremely difficult, because recurrent infections can arise from recrudescence, relapses from hypnozoites or a new infection. Due to the difficulties involved in determining the in vivo treatment failure for *P. vivax* infection, molecular markers seem to be helpful for monitoring and predicting the occurrence of drug resistance in *P. vivax*.

The exact molecular mechanisms of CQ resistance in *P. vivax* remain unclear and it is likely that multigenic loci are involved in this process. *Multidrug resistance 1* (*pvmdr1*) and Plasmodium vivax chloroquine resistance transporter, putative (pvcrt-o), orthologues of genes involved in CQ resistance in *P*. *falciparum*, have been proposed as potential molecular markers; although their exact role in CQ resistance needs to be fully determined [7–10]. The *pvmdr1* gene is located on chromosome 10 and contains 24 single nucleotide polymorphisms (SNPs). Y976F and F1076L mutations in *pvmdr1* have been reported as possible molecular markers of CQ resistance in *P. vivax* [11, 12]. In vitro data showed a significant increase in IC50 value for CQ that correlated with Y976F mutation in *pvmdr1* gene [11]. However, different studies show conflicting results about the role of Y976F and F1076L mutations in CQ resistance. Furthermore, there are some evidences that suggest the involvement of other mechanisms, such as gene amplification and changes in expression levels in the occurrence of CQ resistance in *P. vivax* [13–15]. However, it seems likely that the *pvmdr1* mutations at Y976F and F1076L positions may provide a useful tool to monitor the occurrence and spread of CQ resistance in *P. vivax* due to its variability and spatial patterns. Therefore, surveillance of these potential molecular markers in different endemic areas would be helpful to inform health policy about genetic changes of parasite in response to CQ pressure before the emergence of drug resistance phenotypes.

In 2020, Iran reported zero indigenous cases for the third consecutive year and is considered as malaria eliminated region [16]. However, imported cases from neighbouring countries, including Pakistan and Afghanistan could be the potential source of introducing the drug-resistant strains. The first report of CQ-resistant *P. vivax* in Pakistan was published in 2015 [17]. It could be an alarm signal for malaria control strategies in Iran and highlights the importance of continuous surveillance of the related molecular markers of anti-malarial resistance. Although molecular methods may not be the most effective approach to assess drug resistance in *P. vivax*, reliable molecular markers allow to predict the molecular change of parasite in response to drug pressure before the appearance of resistance phenotype. There is a paucity of studies that have investigated the associated molecular markers with CQ resistance in circulating *P. vivax* isolates from Iran. Therefore, this study was designed to evaluate the current prevalence of Y976F and F1076L mutations in *pvmdr1* gene as the potential markers to monitor CQ resistance in the collected *P. vivax* isolates from Iran. In this study, a simple PCR–RFLP method was developed to assess these mutations and applied different control samples to standardize this molecular analysis.

## Methods

### Study site and samples

Blood samples (n = 120) were collected from *P. vivax* infected patients (2–70 years of age), from Chabahar district, in Sistan and Baluchistan province, Iran. This province is located in the southeastern of Iran bordering Afghanistan and Pakistan with a considerable population movement. The study was conducted at primary health care centers in Chabahar district, where symptomatic malaria patients sought treatment during 2021. Iran is considered a malaria region with zero indigenous cases for the third consecutive year (2018–2020) [16]. All of the studied subjects were imported cases from Pakistan and their mono-infection with *P. vivax* was confirmed by microscopic method. After obtaining an informed consent, thick and thin blood smears were collected from all of the participants. Exclusion criteria were defined as patients who received anti-malarial drugs in the past four weeks and non-consenting individuals. The protocol was in accordance with the ethical standards of the Institutional Review Board of Pasteur Institute of Iran (IR.PII.REC.1395.36) and with the 1964 Helsinki declaration. For molecular diagnosis of *Plasmodium* species, dried blood spots on filter paper were collected by a standard method prior to treatment. All patients with positive microscopy were treated with a standard regimen of CQ plus primaquine for eight weeks and no clinical treatment failure was reported during this period. The collected samples on filter paper were transported to the main laboratory in Pasteur Institute of Iran.

### DNA extraction and species confirmation

*Plasmodium vivax* genomic DNA was extracted from dried blood 3 mm spot by using commercially available DNA purification kit (FAVORGEN, Taiwan) according to the manufacturer's protocol. The extracted DNA was stored at − 20 °C until use. *Plasmodium vivax* infection detected by microscopy in the field was confirmed by PCR in the main laboratory. A nested-PCR amplification of the small sub-unit ribosomal ribonucleic acid (18ssrRNA) gene with one genus-specific (*Plasmodium*) and two species-specific primers (falciparum and vivax) was performed on all samples as described previously [18].

### PCR amplification of *pvmdr1* gene

For amplifying the target sequence of *pvmdr1* gene harbouring putative mutations associated with CQ resistance, primers were designed based on the *pvmdr1* gene sequence (AY618622.1) using Oligo software (Ver. 7.41) (Table 1). Sequence specificity of all designed primers was checked using the primer-BLAST online software (https://www.ncbi.nlm.nih.gov/tools/primer-blast/). PCR amplification of 976 and 1076 positions were performed using the primer combinations F1fla/R1fla and F2fla/R2fla (Table 1), respectively. All PCR reactions for amplification of the target gene were carried out in a final volume of 25 µl, including 2 mM MgCl_2_, 200 µM dNTP mixture (Invitrogen, Carlsbad, CA, USA), 1 unit of Taq DNA polymerase (Invitrogen, Carlsbad, CA, USA), and a pair of primers (10 pmol each). In addition, 150 ng of the extracted DNA was used as a template in the PCR reaction. Negative controls (reaction mixtures without DNA) were included in each amplification reaction. The PCR cycle conditions were as follows: initial denaturation at 94 °C for 5 min, 35 cycles of denaturation at 94 °C for 30 s, annealing at primer-dependent temperature for 30 s, and extension at 72 °C for 30 s, followed by a final extension at 72 °C for 10 min. The PCR products were analysed by electrophoresis on 1.5% agarose gel (Invitrogen, Carlsbad, CA, USA) stained with DNA Green Viewer (Parstous, Mashhad, Iran) and visualized by an ultraviolet light.

**Table 1:**
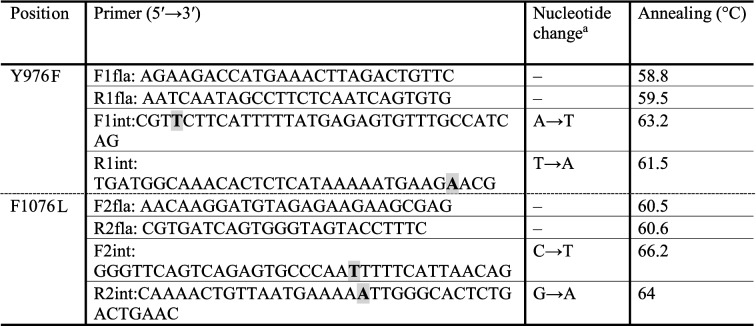
Primers used for molecular analysis of *pvmdr1* gene with their respective annealing temperatures

^a^The changes in the sequence of internal primers used in overlap extension PCR are in bold and gray highlight

### Construction of controls

**Fig. 1:**
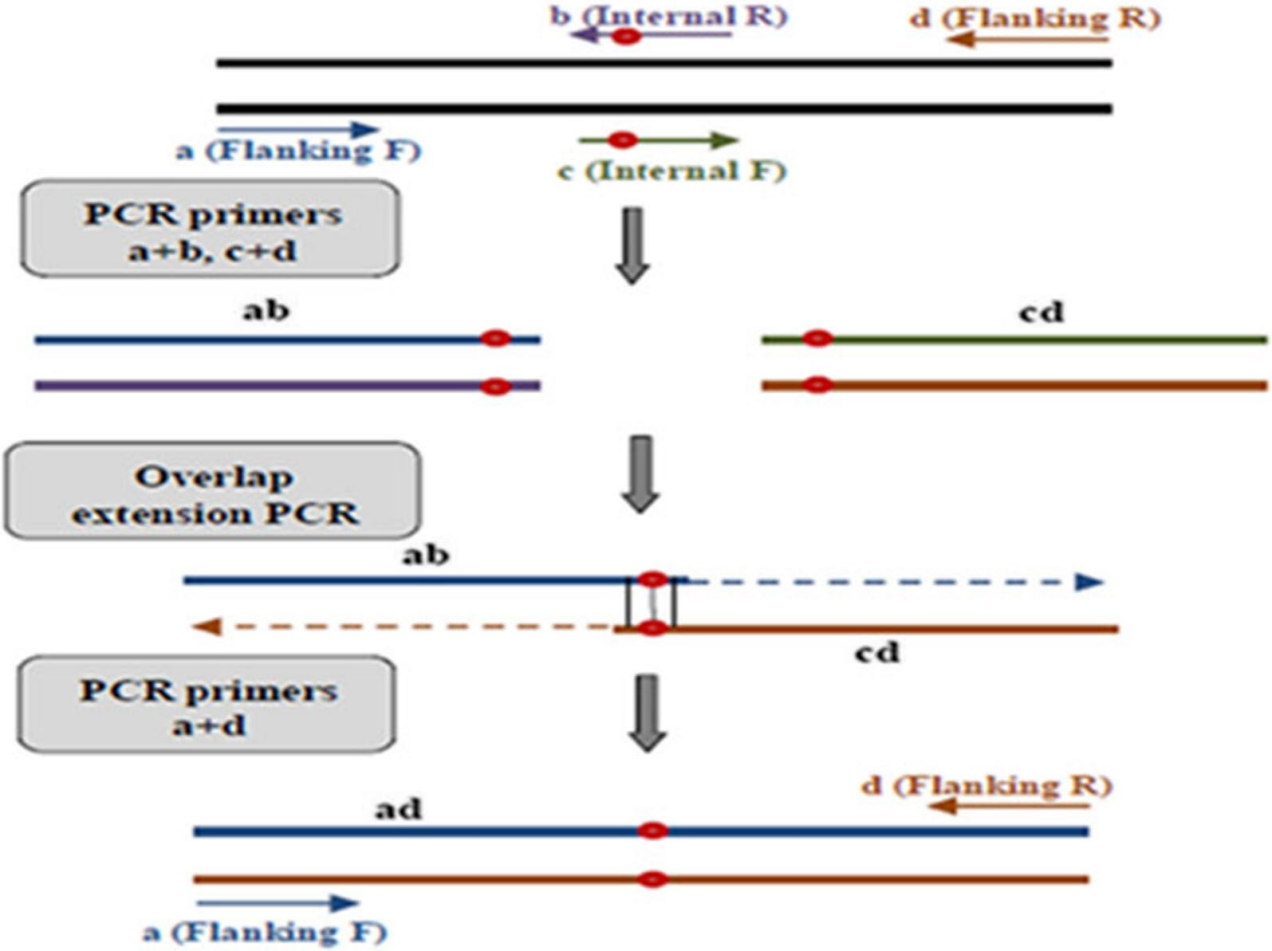
Schematic representation of overlap extension PCR to create specific nucleotide at the target site of *pvmdr1* gene. To construct a mutant control allele for codon 976 and a wild-type control allele for codon 1076 of *pvmdr1* gene, overlap extension PCR was performed. In separate reactions, two overlapping fragments were amplified using Flanking F/Internal R and Internal F/Flanking R primer pairs. The internal primers (Internal F or Internal R) were designed to include the mismatch at 976 or 1076 positions to mimic the mutant or wild-type sequences, respectively. In the second round of PCR reaction, the extension of the overlapped fragments was carried out using Flanking F/Flanking R primer pair

To standardize molecular analysis of *pvmdr1* gene, control samples were first synthesized for the presence and absence of mutations at Y976F and F1076L positions. To construct a wild-type control allele for codon 976, PCR amplification was carried out with the F1fla/R1fla primer pair (468 bp; Table 1) using genomic DNA extracted from a patient blood samples that the absence of Y976F mutation was confirmed by sequencing. To construct a mutated control allele for codon 976, overlap extension PCR was performed as described previously [19]. In summary, two separate PCR reactions were done to amplify two overlapping sub-fragments of the target sequence using F1fla/R1int (270 bp) and F1int/R1fla (231 bp) primer pairs. In each reaction, one flanking primer (F1fla or R1fla) and one internal primer (F1int or R1int) containing the Y976**F** mutation were used. The internal primers were designed to include the mismatch at position 976 to mimic the mutated sequence. The overlapped fragments generated in the first PCRs were gel excised and purified (Qiagen, Hilden, Germany) and their concentrations were determined. Approximately 40 ng of the first reaction products were used in a second round of PCR using F1fla/R1fla primer pair. The extension of the overlap fragments was resulted in a creation of full-length mutant allele (Fig. 1).

To construct wild-type control allele for 1076 codon, overlap extension PCR was done as described for the mutated allele for codon 976. Briefly, in separate reactions, two overlapping fragments were amplified using F2fla/R2int (271 bp) and F2int/R2fla (213 bp) primer pairs. The internal primers (F2int or R2int) were designed to include the mismatch at 1076 position to mimic the wild-type sequence. In the second round of PCR reaction, by using the F2fla/R2fla primer pair (444 bp), the extension of the overlapped fragments was carried out (Fig. 1). For the generation of mutant control allele for codon 1076, PCR amplification was done with the F2fla/R2fla primer pair (444 bp; Table 1) using genomic DNA extracted from a patient blood samples that the presence of F1076**L** mutation was confirmed by sequencing.

The full-length fragments of *pvmdr1* genes containing the desired genotypes were subsequently cloned using the pGEM-T Easy Vector (Promega, Madison, WI). All recombinant plasmids were sequenced to confirm the presence of desired nucleotide at specific position. Subsequently, PCR amplification of control constructs was done using the flanking primer pairs (F1fla/R1fla and F2fla/R2fla for 976 and 1076 positions, respectively). The PCR products of control constructs were used in each RFLP analysis.

### Molecular analysis of *pvmdr1* gene by restriction fragment length polymorphisms (RFLP)

For the analysis of mutations at codons 976 and 1076 of *pvmdr1 *gene, the PCR–RFLP analysis was performed. The mutation at codon 976 creates a restriction site for the enzyme *Mbo*II. However, the mutation at codon 1076 removes a restriction site for the enzyme *Tas*I (*Tsp*EI)*.* Therefore, these enzymes were used for the digestion of the PCR products to define wild and mutant types at Y976F and F1076L positions. RFLP was carried out in 20 µl final volume, including 10 µl PCR products and 10 µl digestion mix containing ddH_2_O, buffer, and enzyme (both from Fermentas, Vilnius, Lithuania) according to the manufacturer’s protocol. The DNA fragments obtained following RFLP were then analysed by electrophoresis on 3% metaphor agarose gels (Lonza Bioscience, Visp, Switzerland). Table 2 shows the expected fragment sizes after the digestion of wild and mutant types at Y976F and F1076L positions.

**Table 2:** The expected fragment sizes after PCR–RFLP for the analysis of mutations at codons 976 and 1076 of the *pvmdr1* gene

Position	Enzyme	Restriction site	Product size (bp)	Incubation °C/(h)
Y976F	*Mbo*II	↓AATT	Y: 154, 150, 150, 14	37 (4)
			F: 154, 150, 81, 69, 14	
F1076L	*Tas*I (*Tsp*EI)	GAAGA(N)8/7↓	F: 151, 124, 92, 43, 34	65 (1)
			L: 151, 124, 92, 77	

## Results

### Confirmation of *Plasmodium vivax* samples

All 120 patients had *P. vivax* mono-infection using both microscopy and nested-PCR and then approved samples were selected for analysis of mutations within the *pvmdr1 *gene.

### Mutation analysis in *pvmdr1* gene

**Fig. 2:**
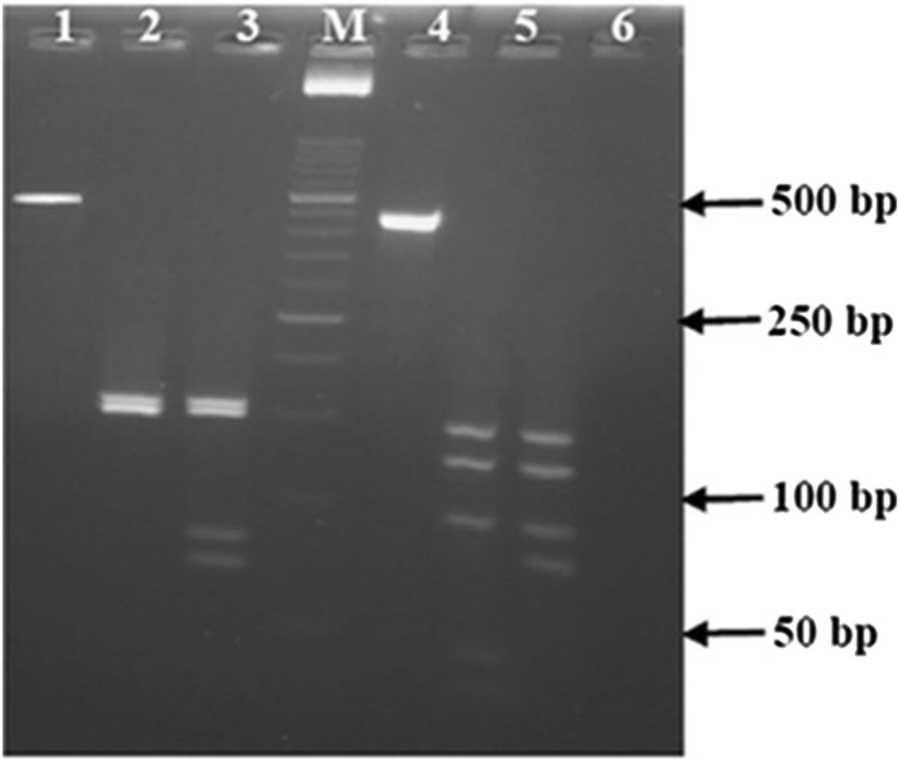
Representative PCR–RFLP patterns from the analysis of codons 976 and 1076 of the *pvmdr1* gene using the *Mbo*II for codon 976 (lanes 1–3) and *Tas*I for codon 1076 (lanes 4–6). Lane 1: undigested PCR product (spanning Y976F position) from control plasmid; Lane 2: *Mbo*II digested PCR product of a wild type control. Lane 3: *Mbo*II digested PCR product of a mutant control sample for Y976F mutation. Lane 4: undigested PCR product (spanning F1076L position) from control plasmid; Lane 5: *Tas*I digested PCR product of a wild type control; Lane 6: *Tas*I digested PCR product of a mutant control sample for F1076L mutation. The lane with the molecular weight marker (50 bp ladder) is labelled M

Of 120 *P. vivax* positive isolates, 105 isolates were analysed for mutations at codons 976 and 1076 of *pvmdr1* gene using RFLP. The obtained DNA fragments following restriction enzyme digestion of the control samples are shown in Fig. 2. Only mutation at codon 1076 in *pvmdr1* gene was observed in *P. vivax* isolates. The F1076L mutation was present in 101/105 (96.2%) samples. In contrast, the Y976F mutation was not found in any of the analysed isolates (Table 3). Only two *pvmdr1* haplotypes were identified, including the single mutant (Y976/1076**L**) as the most prevalent haplotype (with 96.2% frequency) and the wild type (Y976/F1076) with 3.8% frequency (Table 3).

**Table 3:** Frequency distribution of putative mutations and haplotypes of *pvmdr1* gene in 105 *P. vivax* isolates

Gene	*pvmdr1*		No of isolates (%)
Haplotype	Y976F	F1076L	
YF	Y	F	4 (3.8)
Y**L**	Y	**L**	101 (96.2)
**F**F	**F**	F	–
**FL**	**F**	**L**	–

Bold underlined letters denotes the mutant form of amino acids

## Discussion

In Iran, CQ remains generally effective against *P. vivax* [20] and this drug remains the main drug of choice for blood-stage treatment of uncomplicated *P*. *vivax* patients. However, the large influx of imported cases from Pakistan and Afghanistan increases the risk of introducing CQ resistance strains which becomes a major challenge for malaria elimination in Iran. A case report of CQ-resistant *P. vivax* infection in Pakistan [17] may be an emerging threat and support the importance of monitoring drug resistance in endemic regions of Iran bordering Pakistan. To address the potential emergence of CQ-resistant *P. vivax* in imported cases from Pakistan, mutations of candidate resistance marker in *pvmdr1* gene were studied. In this study, a simple PCR–RFLP method was developed and validated to assess molecular markers of CQ resistance in *P. vivax* isolates. A successful elimination programme requires specific tools to monitor the genetic changes in the parasite that could compromise the efficacy of anti-malarial drugs. These tools should be fast, cost-effective, sensitive and easy to perform. PCR–RFLP is a tool that has remarkable potential to detect the emerging mutations of candidate resistance markers.

The current study presents a molecular update on *pvmdr1* gene for more understanding about the possible recent selection of CQ-resistant markers. All of our studied isolates from pre-treatment samples were obtained from patients that were successfully treated with CQ and the result of molecular analysis showed a high proportion of Y976/1076**L** single mutant haplotype (with 96.2% frequency). The earlier molecular study in Hormozgan, Iran showed that most isolates (97/100, 97%) carried single mutant haplotype (Y976/1076**L**) [21], as in the present study (101/105, 96.2%). Compared to *P. vivax* isolates collected in Hormozgan during 2007–2013 [21], there was no evidence for selection of mutants due to CQ pressure. In accordance with these findings, two previous studies from neighbouring Afghanistan [22] and Pakistan [23] revealed that all the isolates were wild type at 976 position and almost all (100% in Afghanistan and 98% in Pakistan) carried the F1076L mutant allele. The dominance of single mutant haplotype in CQ-sensitive isolates in this investigation that is in accordance with previous studies from Iran or bordering countries [21–24] could reflect the fact that *P. vivax* resistance to CQ has not emerged. Some reports suggested the hypothesis of the two-step mutational trajectory in *pvmdr1* gene, with the increase of F1076L mutation followed by Y976F which may lead to CQ resistance [8, 25]. The occurrence of F1076L mutation in almost all the analysed isolates does suggest that resistant genotype will continue to emerge in this region before the appearance of the resistance phenotype. This could potentially provide an early warning that resistance to CQ is possibly emerging. However, the exact role of F1076L mutation in CQ resistance and its clinical significance needs to be determined.

The Y976F mutation in *pvmdr1* gene has been identified as a possible genetic marker of resistance to CQ in Southeast of Asia and the Western Pacific. This mutation almost reached fixation in high-level CQ-resistant *P. vivax* foci including Indonesia and PNG [11, 26]. Conversely, in Thailand with low-grade CQ resistance, prevalence of the Y976**F** mutant allele is at low levels [11, 26]. Additionally, in Korea and India with some rare cases of CQ resistance [27–30], the Y976F mutant was absent in the case of Korea [9, 26, 31] or observed with very low frequency in India [32, 33]. In the case of Eastern Mediterranean regions, where CQ treatment is generally effective, the Y976F mutation was not detected in *P. vivax* isolates from several countries including Iran [21], Pakistan [23] and Afghanistan [22]. Since CQ resistance was also observed in isolates with wild type Y976F [11], this variation in parasite population in different endemic regions may reflect the geographical characteristics of Y976F mutation rather than an association with CQ resistance. This theory has also been suggested previously [21, 34]. Further investigations are needed to assess the role of Y976F mutation in CQ therapeutic failure.

There may be some possible limitations in this study that could be addressed in future research. First, in this study it was not possible to follow up patients for assessment of susceptibility to CQ because all of the analysed samples were collected from Pakistani travellers who returned to their country after receiving treatment. Second, for PCR–RFLP analysis of mutation at codon 976, it was suggested to use *Ac*lI restriction enzyme with the specificity to cut only the Y976**F** mutant allele (reviewer suggestion). However, due to the limitation of this study, the available enzyme (*Mbo*II) was used for mutation analysis of Y976F position. In addition, the relevance of other candidate markers for CQ resistance, including *pvmdr1 *copy number variation, *pvcrt*-*o* gene expression and *pvcrt*-*o* genetic variation cannot be ruled out. Nevertheless, due to the financial issues, the assessment was not possible in the present study. This demands further assessment of the drug resistance markers complemented with in vivo and ex vivo therapeutic efficacy studies to confirm their role in the CQ treatment failure.

## Conclusion

This study provides new data that complements the previous research concerning the molecular characterization of the *P. vivax* isolates that are circulating in Iran. In this study, the absence of Y976F and multiple mutant haplotypes of the *pvmdr1* gene could reflect that resistance to CQ has not emerged in this region. However, the high prevalence of F1076L mutation could potentially provide an early warning that resistant genotype will continue to emerge prior to the appearance of the resistance phenotype. This data is important for performing future epidemiological surveillance to monitor the emergence of CQ resistance in this endemic area and the bordering regions. Continuous monitoring of drug resistance markers complemented with in vivo and ex vivo therapeutic efficacy studies are necessary for proper management and administration of anti-malarial therapies.

## Data Availability

Not applicable.

## Abbreviations

CQ: Chloroquine
*Pvmdr1*: *Plasmodium vivax* *multidrug resistance 1*
*Pvcrt*-*o*: *Plasmodium vivax* *chloroquine resistance transporter, putative*
SNP: Single nucleotide polymorphisms
RFLP: Restriction fragment length polymorphisms
